# Apoptin causes apoptosis in HepG‐2 cells via Ca^2+^ imbalance and activation of the mitochondrial apoptotic pathway

**DOI:** 10.1002/cam4.5528

**Published:** 2022-12-14

**Authors:** Xiaoyang Yu, Tongxing Wang, Yue Li, Yiquan Li, Bing Bai, Jinbo Fang, Jicheng Han, Shanzhi Li, Zhiru Xiu, Zirui Liu, Xia Yang, Yaru Li, Guangze Zhu, Ningyi Jin, Chao Shang, Xiao Li, Yilong Zhu

**Affiliations:** ^1^ Academicians Workstation of Jilin Province Changchun University of Chinese Medicine Changchun China; ^2^ Changchun Veterinary Research Institute Chinese Academy of Agricultural Sciences Changchun China; ^3^ Jiangsu Co‐innovation Center for Prevention and Control of Important Animal Infectious Diseases and Zoonoses Yangzhou China

**Keywords:** apoptin, Ca^2+^ imbalance, endoplasmic reticulum stress, mitochondria apoptosis pathway, mitochondria structural injury

## Abstract

**Background:**

Apoptin is derived from the chicken anemia virus and exhibits specific cytotoxic effects against tumor cells. Herein, we found that Apoptin induced a strong and lasting endoplasmic reticulum (ER) stress response, Ca^2+^ imbalance, and triggered the mitochondrial apoptotic pathway. The aim of this study was to explore the mechanisms by which Apoptin exhibited anti‐tumor effects in HepG‐2 cells.

**Methods:**

The intracellular levels of calcium (Ca^2+^) were induced by ER stress and determined by electron microscopy, flow cytometry, and fluorescence staining. The mitochondrial injury was determined by mitochondrial membrane potential and electron microscopy. Western blotting was used to investigate the levels of key proteins in ER stress and the apoptotic pathway in mitochondria. The relationship between Ca^2+^ levels and apoptosis in Apoptin‐treated cells was analyzed using a Ca^2+^ chelator (BAPTA‐AM), flow cytometry, and fluorescence staining. We also investigated the in vivo effects of Ca^2+^ imbalance on the mitochondrial apoptotic pathway using tumor tissues xenografted on nude mice.

**Results:**

This study showed that Apoptin induced a strong and long‐ lasting ER stress and injury, which subsequently led to an imbalance of cellular Ca^2+^ levels, a reduction in the mitochondrial membrane potential, a significant extent image in the mitochondrial structure, and an increase in the expression levels of Smac/Diablo and Cyto‐C.

**Conclusions:**

In summary, Apoptin induced apoptosis in HepG‐2 cells via Ca^2+^ imbalance and activation of the mitochondrial apoptotic pathway. This study provided a new direction for antitumor research in Apoptin.

## Graphical abstract

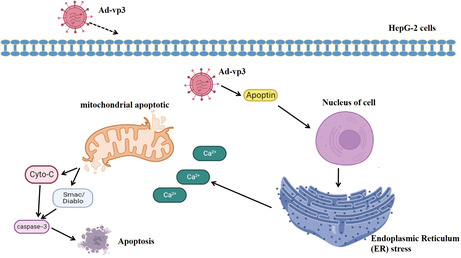

## 1 INTRODUCTION

Apoptin (GenBank: AY171617.1) is derived from chicken anemia virus and exhibits cytotoxicity that is specific to tumor cells.^1^, ^2^ In most of the cell lines investigated thus far, Apoptin has been shown to induce apoptosis in tumor cells via the intrinsic mitochondrial pathway. Apoptin reduces the mitochondrial membrane potential in tumor cells and induces the mitochondria to release cytochrome c (Cyto‐C) and apoptosis‐inducing factor (AIF),^3^, ^4^, ^5^ thus resulting in apoptosis. Apoptin‐induced apoptosis is known to occur independently of p53.^6^ In our research, we found that Apoptin induced a strong and long‐lasting endoplasmic reticulum stress. However, as yet, the specific mechanism underlying the association between ER stress and Apoptin has yet to be elucidated.

The endoplasmic reticulum (ER) is an important intracellular organelle and participates in a number of cellular processes in vivo. The ER is also the main site of calcium (Ca^2+^) storage. Ca^2+^ can be stored in the ER in concentrations of 10–100 mmol/L but is present at concentrations of 100–300 nmol/L in the cytoplasm. A strong and lasting ER stress response creates an imbalance in Ca^2+^. ER stress is known to involve the endoplasmic reticulum kinase (PERK) pathway,^7^, ^8^, ^9^ the inositol‐requiring enzyme1 (IRE1) pathway^10^, ^11^, ^12^ and the activating transcription factor 6 (ATF‐6) pathway.^13^, ^14^

As the key site of energy transformation, the mitochondria also represent the main regulatory site for apoptosis and also play a key role in most of the regulatory processes related to apoptosis. An abnormal change in the mitochondrial membrane potential triggers the release of apoptosis‐related proteins from the mitochondria, including AIF, high‐temperature requirement protein A2 (HtrA2), endonuclease G (Endo G), apoptosis‐related proteins (ARTs), second mitochondria‐derived activator of caspase/direct IAP binding protein with low pl (Smac/Diablo), and cyto‐C. Ca^2+^ imbalance caused by ER stress can affect the structure and function of the mitochondria. Despite the fact that some researches have indicated that Apoptin induces mitochondria to release Cyto‐C and AIF, no previous study has investigated how Apoptin‐induced ER stress can cause Ca^2+^ imbalance and influence the structure and function of the mitochondria. Moreover, previous studies have not investigated the specific effect of Apoptin on the expression of key apoptosis‐related proteins in the mitochondria, such as HtrA2, Smac/Diablo, Endo G, and ATRS.

Therefore, it is of great significance to investigate the specific effect of ER stress on the ER and apoptosis‐related pathways in the mitochondria with regard to the induction of apoptosis. In the present study, we demonstrated that Apoptin can induce ER stress, cause Ca^2+^ imbalance, affect the structure and function of the mitochondria, and induce significant changes in the expression of key proteins related to the ER stress and the mitochondria. Our research involved the assessment of dynamic changes in the ER and mitochondria, the determination of Ca^2+^ levels, western blotting, and immunohistochemical analysis of tumor tissue from a tumor‐bearing nude mouse model.

## 2 MATERIALS AND METHODS

### 2.1 Materials

The human hepatoma cell line HepG‐2 was purchased from the Cell Bank of the Chinese Academy of Sciences (Shanghai, China). We also used a recombinant type 5 adenovirus (Ad‐Apoptin; hereafter referred to as Ad‐Vp3, 1 × 10^8^ PFU) that contained a CMV promoter and the Apoptin gene from the chicken anemia virus. We used the recombinant type 5 adenovirus (hereafter referred to as Ad‐Mock, 1 × 10^8^ PFU) without the Apoptin gene as a blank control. Ad‐Vp3 and Ad‐Mock were constructed previously in our laboratory.

Six‐week‐old male BALB/c nude mice were housed in a controlled room (temperature: 22 ± 2°C; humidity: 50 ± 5%) and provided with a solid diet (Changchun Billion Adams Laboratory Animal Technology Co., Ltd.) and sterilized tap water. All mice were obtained from Beijing vital River Laboratory Animal Technology Co., Ltd., and the animal experiment protocols were approved by the Institutional Animal Care and Use Committee (IACUC) of the Changchun University of Chinese Medicine. All surgical procedures were performed under 40 mg/kg 1% sodium pentobarbital via intraperitoneal injection.

Dihydrorhodamine 123 (DHR) (No. D632), JC‐1 (No. T3168), MitoTracker™ Red CMXRos (TMRM) (No. M7512), Oregon Green™ 488 BAPTA‐1, AM, and Cell‐permeant Special Packaging (No. O6807) were purchased from ThermoFisher Scientific (Shanghai, China). We also purchased a FITC Annexin V Apoptosis Detection Kit I (No. 556547) from BD Biosciences (Beijing, China). We also acquired a Minute™ Total Protein Extraction Kit (No. SD‐001/SN‐002) and a Mitochondria Isolation Kit For Mammalian Cells And Tissues (No. MP‐007) from Invent (Beijing, China). We purchased a range of reagents from Cell Signaling Technology (Shanghai, China), including a PARP (46D11) Rabbit mAb (No. 9532), Caspase‐3 (D3R6Y) Rabbit mAb (No. 14220), Cytochrome c (D18C7) Rabbit mAb (No. 11940), Smac/Diablo (D5S3R) Rabbit mAb (No. 15108), Endonuclease G Antibody (No. 4969). We also purchased a monoclonal anti‐ARTS antibody mAb (No. 106M4840) from Sigma‐Aldrich (Shanghai, China). We purchased an anti‐AIF antibody (No. ab1998), anti‐HtrA2/Omi antibody (No. ab75982), anti‐PERK antibody (No. ab65142), anti‐ATF‐6 (ab37149), and anti‐IRE1 (ab37073) from Abcam (Shanghai, China).

### 2.2 Methods

#### 2.2.1 Determination of virus titer

The virus titer was determined as previously described.^15^ In brief, viruses were serially diluted in a serum‐free DMEM growth medium. Ten‐fold dilutions were added to 96‐well plates in a volume of 100 ml/well. HEK293 cells were seeded into the plates 1 day before at a density of 5 × 10^3^/well. Next, 100 ml of DMEM with 4% FBS, 100 IU/ml of penicillin, and 100 mg/ml of streptomycin were added 2 h post‐infection to reach a final volume of 200 ml per well. Cells were then cultured at 37°C with 5% CO_2_ for 4 days. Viral titers were calculated using the Reed‐Muench method in the form of TCID_50_.

#### 2.2.2 Annexin V analysis

HepG‐2 cells were seeded at a density of 6 × 10^5^ cells per well in a 6‐well plate and incubated for 24 h at 37°C in 5% CO_2_. Cells were then infected with Ad‐Vp3 (MOI: 50) for 12, 24, 36, 48, and 72 h. Ad‐Vp3‐infected HepG‐2 cells were incubated in the dark for 15 min at room temperature in the presence of 5 μl Annexin V‐FITC and 5 μl of PI in 100 μl of 1 × binding buffer. We then quantified the extent of apoptosis in HepG‐2 cells by flow cytometry. Ad‐Mock‐infected cells and non‐infected HepG‐2 cells were used as negative controls.

#### 2.2.3 Analysis of ROS levels

HepG‐2 cells were seeded at 6 × 10^6^ cells per well in a 6‐well plate and incubated for 24 h at 37°C with 5% CO_2_. The cells were then infected with 50 MOI Ad‐Vp3 for 12, 24, 36, 48, and 72 h. HepG‐2 cells infected with Ad‐Vp3 were incubated in the dark for 15 min at room temperature in the presence of 1 μl DHR in 1 ml of DMEM. Flow cytometry was used to detect the levels of ROS in HepG‐2 cells infected with Ad‐Vp3. Ad‐Mock‐infected cells and non‐infected HepG‐2 cells were used as negative controls.

#### 2.2.4 Electron microscopy

HepG‐2 cells were seeded at a density of 6 × 10^5^ cells/well into a 6‐well plate, cultured for 24 h at 37°C in 5% CO_2_, and infected with Ad‐Vp3 (MOI: 50) for 48 h. Cells were then harvested and washed with PBS and sections were prepared using ultramicrotomy. Cells were analyzed by electron microscopy. Ad‐Mock‐infected cells and non‐infected HepG‐2 cells were used as negative controls.

#### 2.2.5 Analysis of Ca^2+^ levels

HepG‐2 cells were seeded at a density of 6 × 10^5^ cells/well into a 6‐well plate, cultured for 24 h at 37°C in 5% CO_2_, and infected with Ad‐Vp3 (MOI: 50) for 12, 24, 36, 48, and 72 h. Cells were then incubated in the dark for 20 min at 37°C in the presence of 1 μg Oregon Green™ 488 BAPTA‐1. Then, we quantified Ca^2+^ levels in the HepG‐2 cells by fluorescence microscopy and flow cytometry, in accordance with the manufacturer's instructions. Ad‐Mock‐infected cells and non‐infected HepG‐2 cells were used as negative controls.

#### 2.2.6 Mitochondrial analysis: structure and function

HepG‐2 cells were seeded at a density of 6 × 10^5^ cells/well into a 6‐well plate, cultured for 24 h at 37°C in 5% CO_2_, and infected with Ad‐Vp3 (MOI: 50) for 12, 24, 36, 48, and 72 h. Cells were then incubated in the dark for 20 min at 37°C in the presence of 1 μl JC‐1 in 1 ml of DMEM. Then, we determined the mitochondrial membrane potential of HepG‐2 cells by flow cytometry. Ad‐Mock‐infected cells and non‐infected HepG‐2 cells were used as negative controls.

For structural analysis, HepG‐2 cells were seeded at a density of 1 × 10^7^ cells into a 100 mm cell culture dish, cultured for 24 h at 37°C in 5% CO_2_ and infected with Ad‐Vp3 (MOI: 50) for 48 and 72 h. Then, we collected mitochondria with a Mitochondria Isolation Kit for Mammalian Cells and Tissues, in accordance with the manufacturer's guidelines. Then, we prepared sections by ultramicrotomy and investigated the structure of the mitochondria by electron microscopy Ad‐Mock‐infected cells; non‐infected HepG‐2 cells were used as negative controls.

#### 2.2.7 Western blotting

Western blot analysis was performed as previously described.^16^, ^17^ HepG‐2 cells were seeded at a density of 6 × 10^5^ cells/well into a 6‐well plate, cultured for 24 h at 37°C in 5% CO_2_ and infected with Ad‐Vp3 (MOI: 50) for 12, 24, 36, 48, and 72 h. Ad‐Mock‐infected cells were used as negative controls. Total protein was extracted from cells using the Minute™ Total Protein Extraction Kit, separated by SDS‐PAGE, and transferred to membranes for western blotting so that we could determine the expression levels of proteins related to apoptotic proteins, ER stress pathway, and apoptotic pathways in the mitochondria.

#### 2.2.8 Analysis of Ca^2+^ imbalance

HepG‐2 cells were seeded at a density of 6 × 10^5^ cells/well into a 6‐well plate, cultured for 24 h at 37°C in 5% CO_2_, and treated with Ad‐Vp3 (MOI: 50) and 10 μM BAPTA‐AM for 72 h. For Annexin V analysis, the cells were incubated in the dark for 15 min at room temperature in the presence of 5 μl Annexin V‐FITC and 5 μl of PI in 100 μl of 1 × binding buffer. Then, we quantified the extent of apoptosis in HepG‐2 cells by fluorescence microscopy and flow cytometry. For mitochondrial analysis, cells were incubated in the dark for 20 min at 37°C in the presence of 1 μl of JC‐1 in 1 ml of DMEM. Then, we determined the mitochondrial membrane potential of HepG‐2 cells by fluorescence microscopy and low cytometry.

#### 2.2.9 In vivo studies

The xenograft models were established by the subcutaneous injection of HepG‐2 cells (3 × 10^6^/100 μl) with Matrigel® Matrix Basement Membrane (yielding a 1:1 ratio) into the left leg of each mouse. When the tumors had formed clearly (usually 14 days), the mice were divided randomly into five groups (9 mice per group). Group 1 was injected with 1 × 10^8^ plaque‐forming units (PFU) of Ad‐Vp3 and 5 mg/kg BAPTA‐AM and group 2 was injected with Ad‐Vp3 (1 × 10^8^ PFU). Group 3 was injected with 5 mg/kg BAPTA‐AM. Group 4 was injected with Ad‐Mock (1 × 10^8^ PFU) and group 5 was injected with the same volume of vehicle. All groups were infected with Ad‐Vp3 or Ad‐Mock via intra‐tumoral injection and treated with BAPTA‐AM via intra‐peritoneal injection from day 14. The injections were given every 3 days for 15 days. Tumor volumes (length × width^2^ × 0.5) and survival rates were calculated every 7 days for 35 days. During the experiment, tumors from three mice (from each group) were harvested. Then, we used western blotting, along with histopathological and immunohistochemical staining, to investigate the apoptotic pathway in mitochondria, including AIF, HtrA2, Smac/Diablo, and Cyto‐C.^18^ All immunohistochemical detection was performed by Servicebio (Wuhan, China). The TUNEL assay was used to measure Ad‐Vp3 + BAPTA‐AM and Ad‐Vp3 group tumors^19^, ^20^. All experimental procedures involving animals were approved by the Institutional Animal Care and Use Committee of the Changchun University of Chinese Medicine.

### 2.3 Statistics

All statistical analyses were performed by using the Statistical Package for the Social Sciences (SPSS) statistical software package (version 15.0; SPSS Inc., Chicago, IL, USA). Data were presented using GraphPad Prism version 7.0 (GraphPad Software Inc., La Jolla, CA, USA). For analysis, we used the Student's t‐test or one‐way analysis of variance (ANOVA) followed by Tukey's post hoc test; *p* < 0.05, *p* < 0.01, or *p* < 0.001, were considered to be statistically significant. Data are presented as the mean ± standard deviation (SD).^21^

Immunohistochemical analyses were performed using Image‐Pro Plus 6.0 software (Media Cybernetics, Inc., Rockville, MD, USA). At least three fields of vision were randomly selected for each slice (and for each group). When acquiring images, we organized the entire field of view to ensure that the background illumination was consistent. A method was used to define positive staining in cells for other types of proteins; brown/yellow staining indicated positive immunostaining. We used Image‐Pro Plus 6.0 software to determine the integrated optical density (IOD) and the area occupied by the entire tissue (AREA). The areal density was then defined by IOD/AREA. A larger areal density indicated a higher level of positive expression.

## 3 RESULTS

### 3.1 Apoptosis was induced in HepG‐2 cells by Apoptin

**FIGURE 1:**
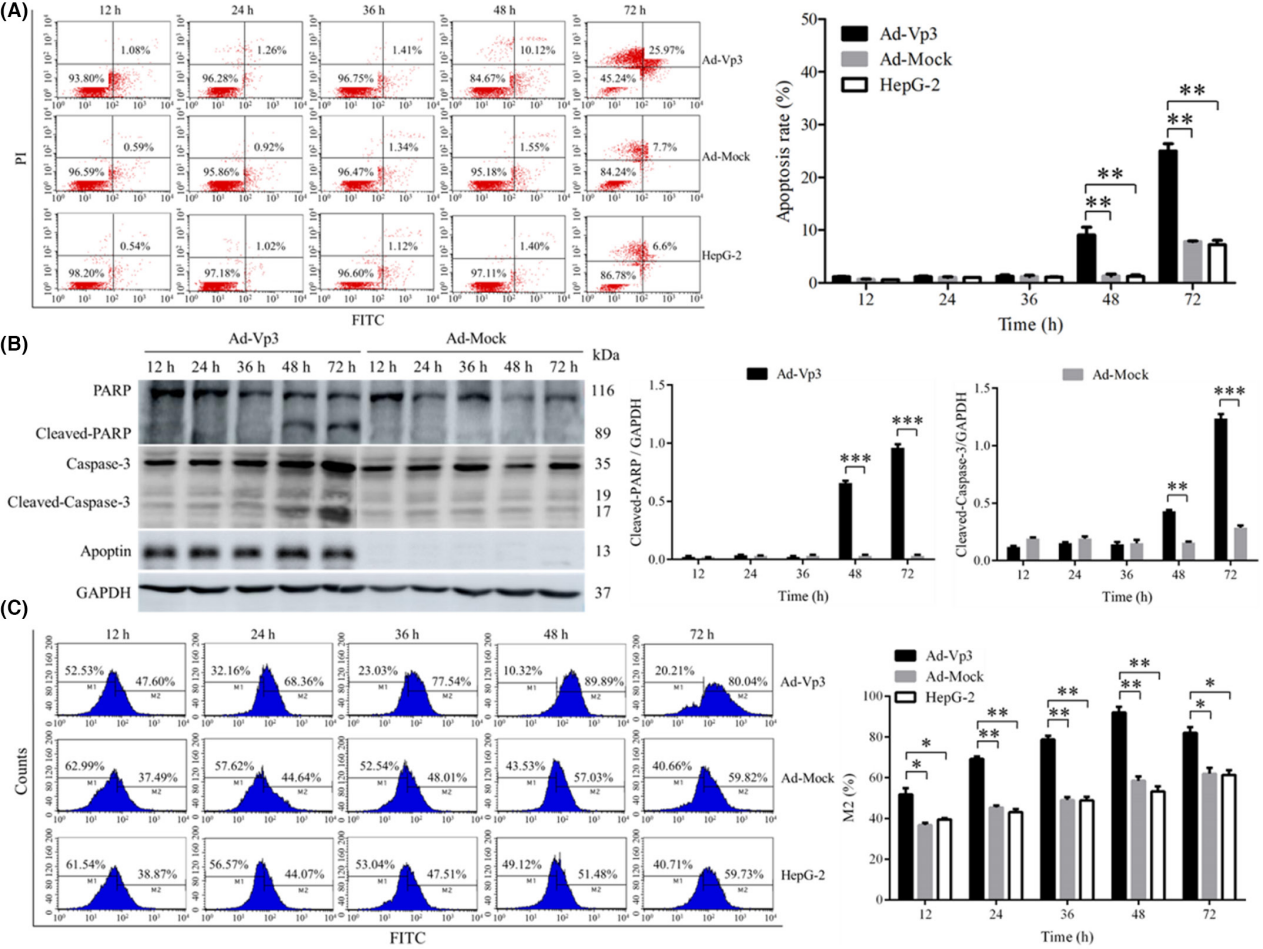
The apoptotic effects of Apoptin on HepG‐2 cells. Apoptin significantly induced apoptosis in HepG‐2 cells. (A) The detection of Annexin V‐FITC/PI by flow cytometry in Ad‐Vp3‐infected HepG‐2 cells. The apoptosis level of HepG‐2 cells infected with Ad‐Vp3 was significantly higher at 48 and 72 h. (B) Western blot analysis of apoptosis‐related proteins PARP and Caspase‐3. The levels of Cleaved‐PARP and Cleaved‐caspase‐3 in the Ad‐Vp3 group were higher than those in Ad‐Mock at 48 and 72 h. (C) The intracellular ROS was detected by flow cytometry. The level of ROS in the Ad‐Vp3 group was significantly higher than that in Ad‐Mock‐infected cells and non‐infected HepG‐2 cells at 12, 24, 36, 48, and 72 h post‐infection. Data are presented as mean ± SD, *p < 0.05, **p < 0.01, ***p < 0.001.

Apoptosis in HepG‐2 cells was investigated by flow cytometry, the detection of proteins related to apoptosis, and the detection of ROS levels. Apoptosis in HepG‐2 cells was induced significantly by Apoptin (Figure 1A). The apoptotic rate in Ad‐Vp3‐infected cells was significantly higher than that in Ad‐Mock‐infected cells and non‐infected HepG‐2 cells at 48 and 72 h post‐infection (*p* < 0.05). Data related to apoptosis‐related proteins in Ad‐Vp3‐infected HepG‐2 cells are shown in Figure 1B. The expression levels of Cleaved‐PARP and Cleaved‐Caspase‐3 in Ad‐Vp3‐infected HepG‐2 cells were significantly higher at 48 and 72 h post‐infection when compared with HepG‐2 cells infected with Ad‐Mock (*p* < 0.01). The ROS levels of HepG‐2 cells were significantly induced by Apoptin (Figure 1C). The levels of ROS in Ad‐Vp3‐infected cells were significantly higher than that in Ad‐Mock‐infected cells and HepG‐2 cells at all time points (*p* < 0.05).

### 3.2 Apoptin induction of endoplasmic reticulum stress

**FIGURE 2:**
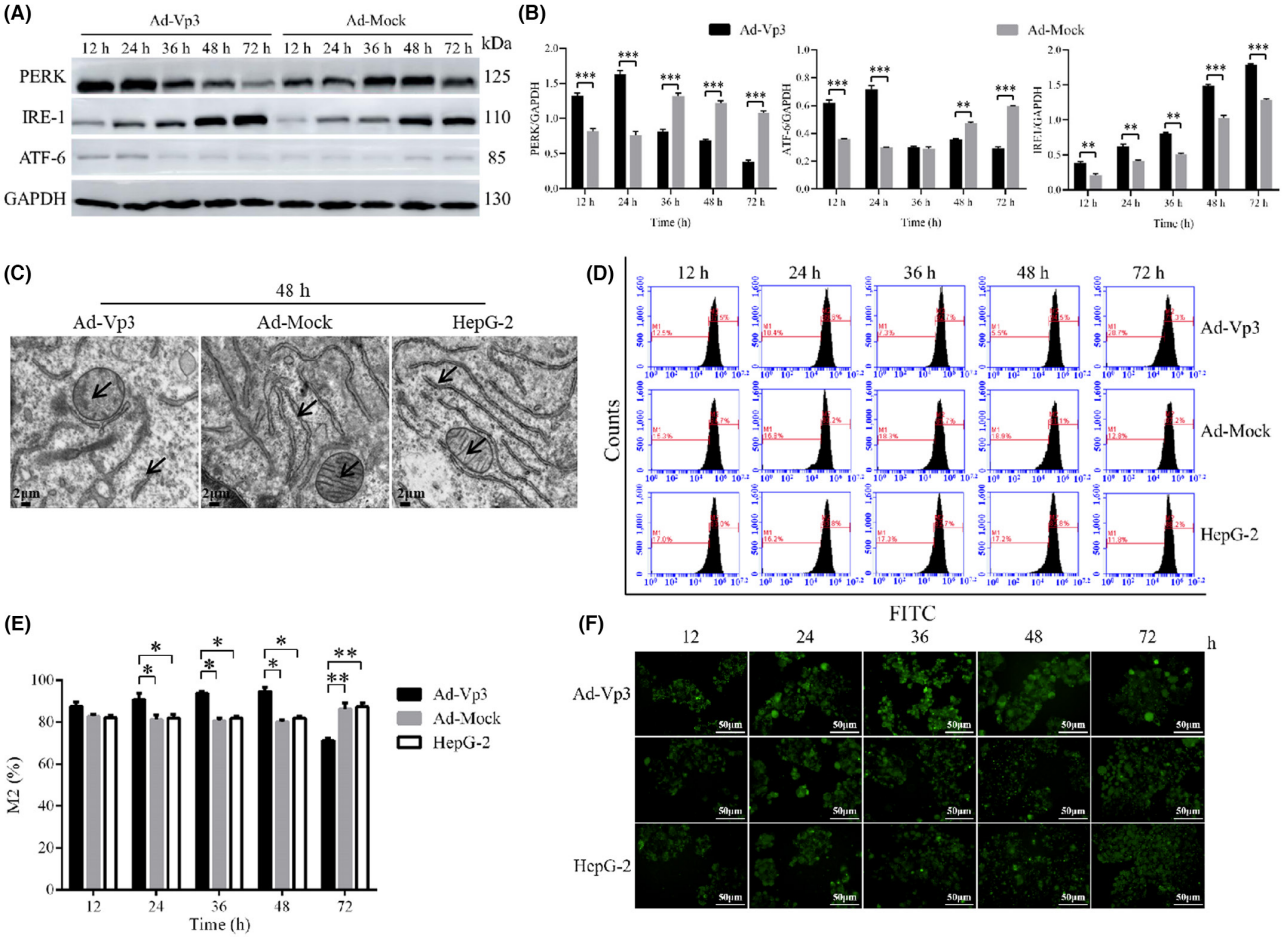
The effect of ER stress injury on HepG‐2 cells. (A, B) Western blot analysis of the expression levels of proteins related to the ER stress pathway in ER. Apoptin triggered the ER stress pathway, which could increase the expression of PERK, IRE1, and ATF‐6. (C) The structure of the ER in Ad‐Vp3‐infected HepG‐2 cells was determined by electron microscopy at 48 h post‐infection (5000×). The arrows represent ER and the mitochondria. The structure of the ER and the crest of the mitochondria in Ad‐Vp3‐infected cells had incurred damage at 48 h post‐infection. (D, E) Flow cytometry detection of ER using Oregon Green™ 488 BAPTA‐1, AM, cell permeant‐Special Packaging. (F) Endoplasmic reticulum Oregon Green™ 488 BAPTA‐1, AM, cell permeant‐Special Packaging staining (200×). Data are presented as mean ± SD, *p < 0.05, **p < 0.01.

The effect of Apoptin on ER stress in HepG‐2 cells was investigated by detecting ER stress‐related proteins. Our analysis showed that some of the proteins related to the ER stress pathway were significantly affected by Apoptin (Figure. 2A, B). For example, the expression of PERK and ATF‐6 in Ad‐Vp3‐infected cells were significantly higher at 12 and 24 h than in cells infected with Ad‐Mock but were significantly lower at 48 and 72 h post‐infection (*p* < 0.01). The expression level of IRE1 in Ad‐Vp3‐infected cells was significantly higher than that in Ad‐Mock‐infected cells at all time points (*p* < 0.01).

The damage caused by apoptin to the endoplasmic reticulum and mitochondria was determined by electron microscopy. The effect of Apoptin on the structure of the ER was evaluated by electron microscopy. Electron microscopy revealed clear structural damage in the ER and mitochondria of infected cells at 48 h (Figure 2C). The structure of the ER and the crest of the mitochondria in Ad‐Vp3‐infected cells had incurred significant damage by 48 h post‐infection. This result indicated that the strong and lasting ER stress response can damage the structure of the ER.

The effect of apoptin on Ca^2+^ levels was determined by fluorescence staining and flow cytometry. The intensity of green fluorescence (as an indicator of Ca^2+^ level) in HepG‐2 cells infected with Ad‐Vp3 was significantly higher than cells infected with Ad‐Mock at 24, 36, and 48 h post‐infection. At 72 h post‐infection, the level of green fluorescence in the HepG‐2 cells infected with Ad‐Vp3 was significantly lower than that in the control group (Figure 2D, E) (*p* < 0.05). The trend for green fluorescence over time was similar to that shown by flow cytometry (Figure 2F). These results showed that ER stress increased the levels of Ca^2+^, thus leading to an imbalance in Ca^2+^. Therefore, Apoptin can induce a strong and lasting ER stress that ultimately leads to Ca^2+^ imbalance.

### 3.3 Effect of Apoptin on mitochondria

**FIGURE 3:**
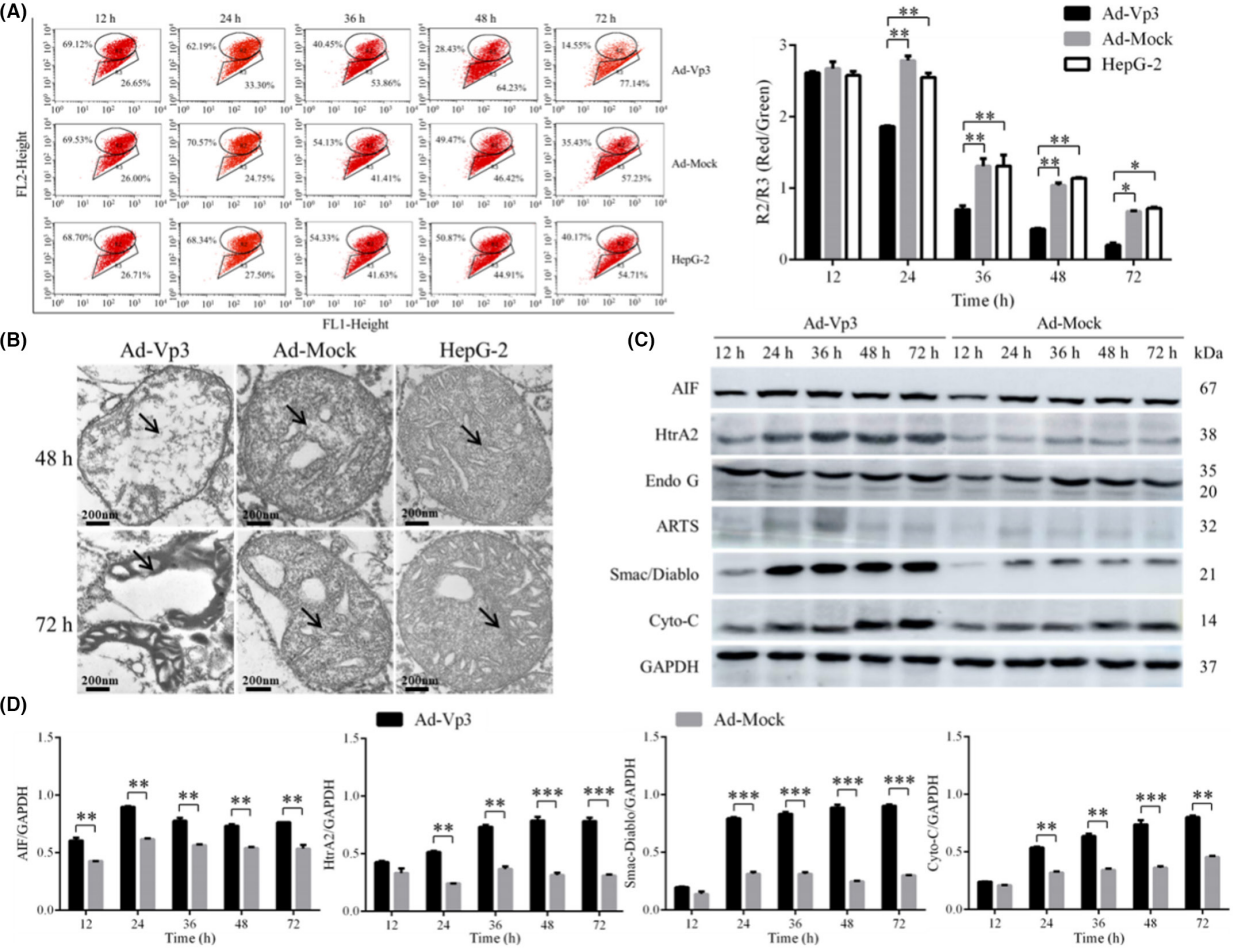
Apoptin significantly reduced mitochondrial membrane potential and caused damage to the mitochondrial structure. (A) Mitochondrial membrane potential, as determined by JC‐1 and flow cytometry. (B) The structure of the mitochondria, as observed by electron microscopy at 48 and 72 h post‐infection (30000×). The arrows represent mitochondria. The crest of the mitochondria in Ad‐Vp3‐infected cells had been broken and dissolved at 48 h post‐infection. The mitochondria had been destroyed at 72 h post‐infection. (C, D) Western blot analysis of the expression of proteins related to the apoptotic pathway in the mitochondria. Apoptin triggered the mitochondrial apoptotic pathway to increase the expression levels of AIF, HtrA2, Smac/Diablo, and Cyto‐C. Data are presented as mean ± SD, *p < 0.05, **p < 0.01, ***p < 0.001.

The effect of apoptin on mitochondrial membrane potential was detected by flow cytometry. The mitochondrial membrane potential of HepG‐2 cells was significantly reduced by Apoptin. The relative fluorescence (R2/R3; Red/Green) associated with mitochondrial membrane potential in Ad‐Vp3‐infected HepG‐2 cells was significantly lower than that in Ad‐Mock‐infected cells and non‐infected cells at 24, 36, 48, and 72 h post‐infection (Figure 3A) (*p* < 0.05).

The mitochondrial damage caused by apoptin was determined by electron microscopy. It was also evident that Apoptin caused damage to the mitochondria of HepG‐2 cells (Figure 3B); the crest of the mitochondria in Ad‐Vp3‐infected cells appeared to be broken and dissolved at 48 h post‐infection. At 72 h post‐infection, it was evident that the mitochondria had been completely destroyed.

By detecting mitochondrial apoptosis‐related proteins, it was clear that Apoptin can cause apoptosis of HepG‐2 cells through the mitochondrial apoptosis pathway. The expression levels of AIF, HtrA2, Smac/Diablo, and Cyto‐C in Ad‐Vp3‐infected cells were significantly higher than those in Ad‐Mock‐infected cells at all time points (Figure 3C, D) (*p* < 0.05). However, there were no significant differences with regard to the expression of ARTs and Endo G in HepG‐2 cells infected with Ad‐Vp3 and Ad‐Mock (*p* > 0.05). It was evident that Apoptin can trigger the apoptotic pathway in the mitochondria by increasing the expression levels of AIF, HtrA2, Smac/Diablo, and Cyto‐C.

Collectively, these results indicated that Apoptin significantly reduced mitochondrial membrane potential, caused damage to the mitochondrial structure, and triggered the apoptotic pathway in the mitochondria of HepG‐2 cells.

### 3.4 BAPTA‐AM reduced the apoptosis in HepG‐2 cell induced by Apoptin

**FIGURE 4:**
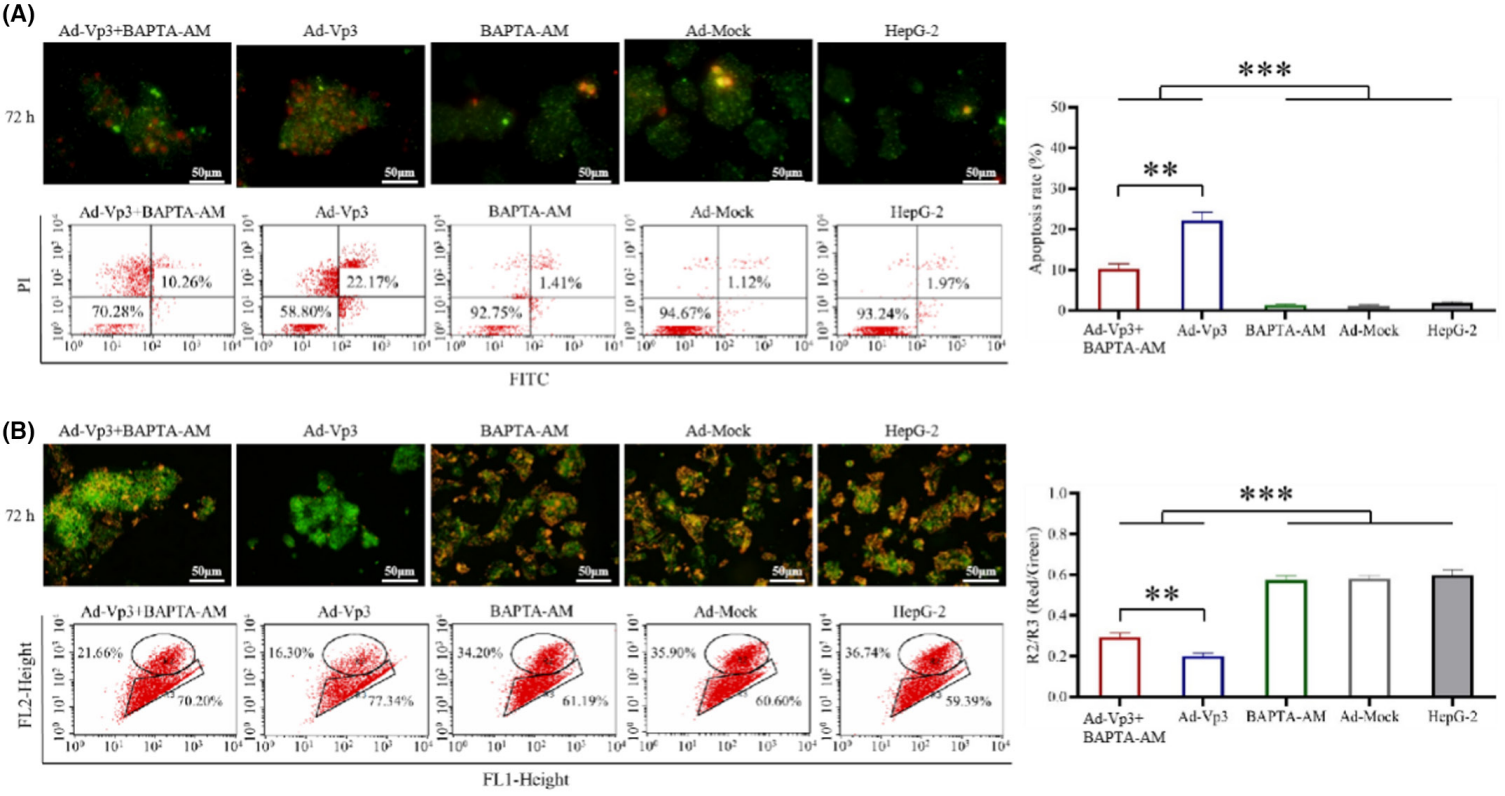
Relationship between Ca^2+^ level and apoptosis in HepG‐2 cells infected with Ad‐Vp3. (A) The detection of Annexin V‐FITC/PI by flow cytometry and fluorescence staining at 72 h post‐infection (200×). (B) Mitochondrial membrane potential, as determined by JC‐1 flow cytometry and fluorescence staining at 72 h post‐infection (200×). Data are presented as mean ± SD, **p < 0.01, ***p < 0.001.

To verify whether Ca^2+^ imbalance has an effect on HepG‐2 cells, we performed Annexin V and JC‐1 analysis on Ad‐Vp3‐infected HepG‐2 cells (Figure 4A, B). The apoptotic rate in Ad‐Vp3 + BAPTA‐AM‐treated cells and Ad‐Vp3‐infected cells was significantly higher than that in BAPTA‐AM‐treated cells, Ad‐Mock‐infected cells, and HepG‐2 cells at 72 h post‐infection (*p* < 0.001), while that in Ad‐Vp3 + BAPTA‐AM‐treated cells was significantly lower than that in Ad‐Vp3‐infected cells at 72 h post‐infection (Figure 4A) (*p* < 0.01). The mitochondrial membrane potential of HepG‐2 cells in the Ad‐Vp3 + BAPTA‐AM group and Ad‐Vp3 group was significantly higher than that in the BAPTA‐AM group, Ad‐Mock group, and HepG‐2 group at 72 h post‐infection (*p* < 0.001), while that in Ad‐Vp3 + BAPTA‐AM group was significantly higher than that in Ad‐Vp3 group at 72 h post‐infection (Figure 4B) (*p* < 0.01). These results showed that apoptotic rate and mitochondrial membrane potential were significantly affected by Ca^2+^ imbalance.

### 3.5 Ca^2+^ level affected the antitumor ability of Apoptin in vivo

**FIGURE 5:**
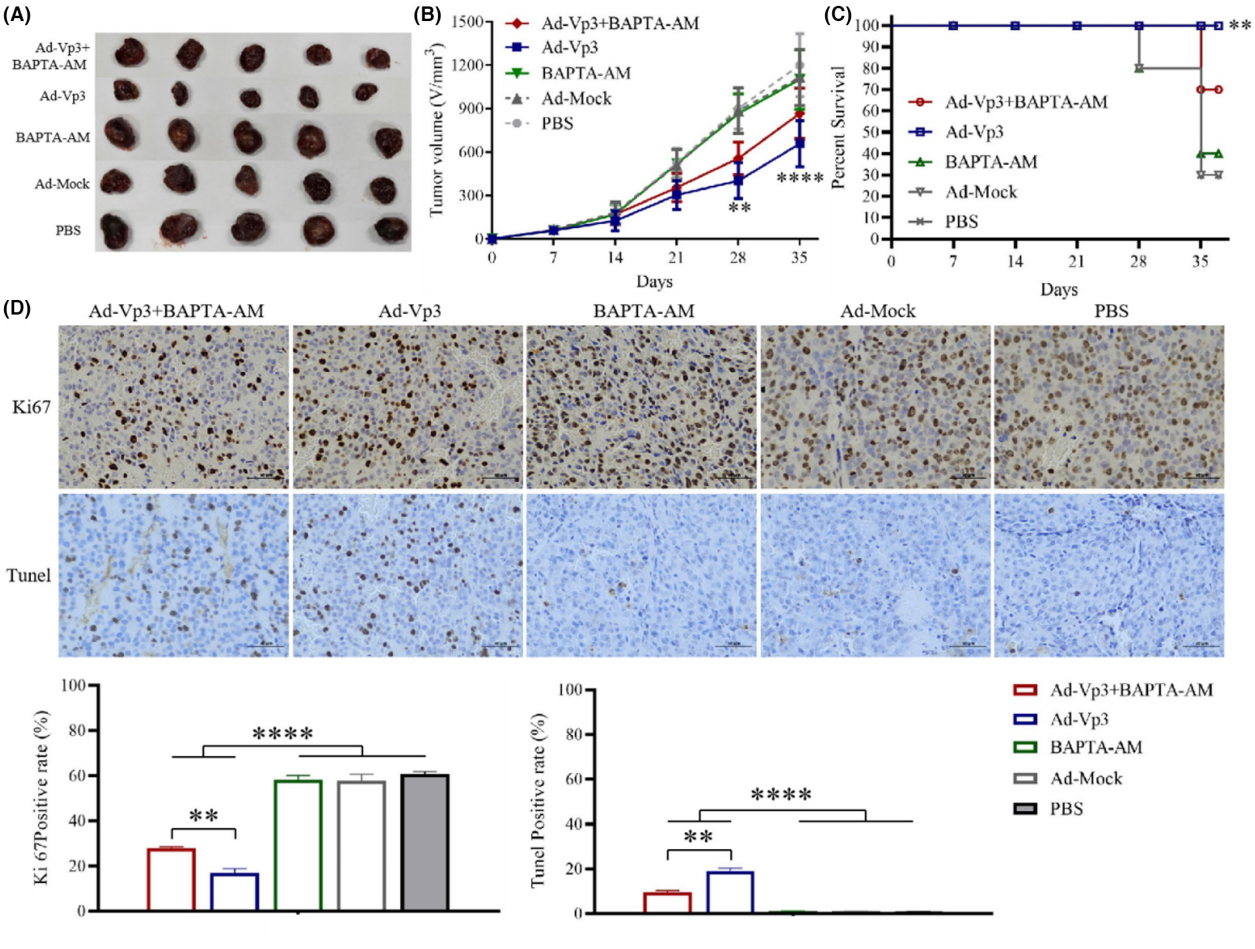
Inhibitory effect of Ad‐Vp3 + BAPTA‐AM on HepG‐2 cells in vivo. Apoptin significantly inhibits tumor growth and prolongs the survival of tumor‐bearing nude mice, while BAPTA‐AM significantly inhibited the antitumor effect of Apoptin. (A, B) The analysis of tumor volume post‐virus infection. (C) The analysis of survival rate post‐virus infection. (D, E) Ki67 staining and TUNEL analysis of tumor tissues (400×). Data are presented as mean ± SD, *p < 0.05, **p < 0.01.

The effect of calcium imbalance on Apoptin's anti‐liver cancer was determined by using a xenograft tumor model. The antitumor effects of Ad‐Vp3 can be significantly reduced by BAPTA‐AM in the HepG2‐xenografted nude mice. The tumor volume in the Ad‐Vp3 + BAPTA‐AM group was significantly lower than that in the Ad‐Vp3 group, at 28 and 35 days after tumor xenografting (Figure 5A, B) (*p* < 0.01). The survival rate of the Ad‐Vp3 group was 100%, while that of the Ad‐Vp3 + BAPTA‐AM group was 70% when evaluated 35 days after tumor xenografting. The survival rate in the Ad‐Vp3 group was significantly longer than that in the Ad‐Vp3 + BAPTA‐AM group (Figure 5C) (*p* < 0.01). The Ki67positivity rate in the Ad‐Vp3 + BAPTA‐AM and Ad‐Vp3 group tumors was significantly lower than that in the BAPTA‐AM, Ad‐Mock, and PBS groups (*p* < 0.0001) while that in the Ad‐Vp3 + BAPTA‐AM group was significantly higher than that in the Ad‐Vp3 group (*p* < 0.01). The TUNEL positivity rate in the Ad‐Vp3 + BAPTA‐AM and Ad‐Vp3 group tumors was significantly higher than that in the BAPTA‐AM, Ad‐Mock, and PBS groups (*p* < 0.0001), while that in Ad‐Vp3 + BAPTA‐AM group was significantly lower than that in Ad‐Vp3 group (Figure 5D) (*p* < 0.01). These results showed that BAPTA‐AM could significantly inhibit the antitumor effect of Apoptin.

### 3.6 Western‐blot and immunohistochemical detection of mitochondrial proteins related to the apoptotic pathway in tumor tissues

**FIGURE 6:**
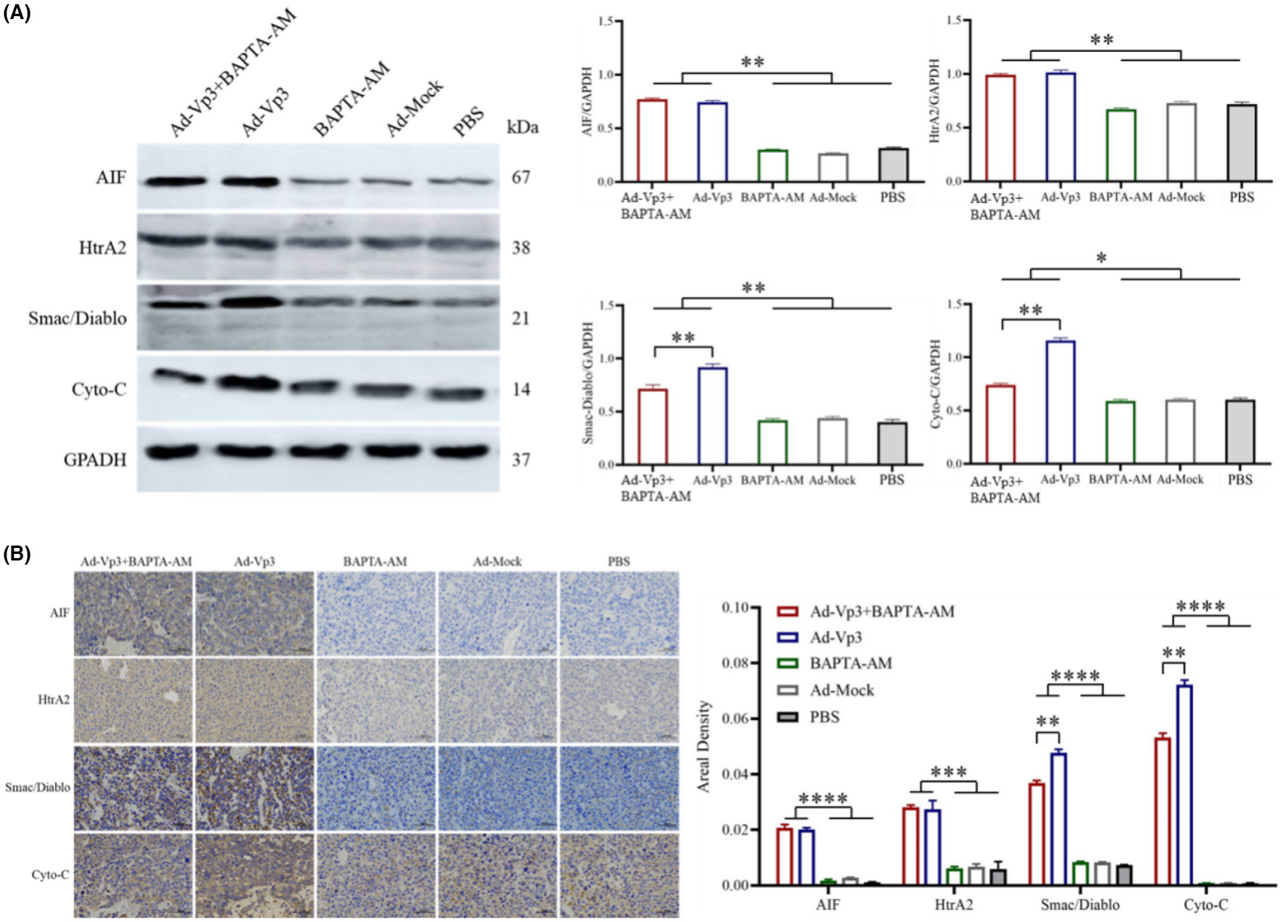
The western blot and immunohistochemical detection of apoptotic proteins related to the mitochondria. The expression of proteins related to apoptotic pathways in the mitochondria (AIF, HtrA2, Smac/Diablo, Cyto‐C), as detected by western blot (A) and immunocytochemistry (B) (400×). Apoptin significantly induced the expression levels of AIF, HtrA2, Smac/Diablo, and Cyto‐C, while BAPTA‐AM significantly reduced the expression of Smac/Diablo and Cyto‐C in Apoptin‐treated tumor tissues. Data are presented as mean ± SD, *p < 0.05, **p < 0.01, ***p < 0.001, ****p < 0.0001.

Western blotting and immunohistochemical assays were used to investigate the effects of changes in calcium ion levels on proteins associated with the mitochondrial apoptosis pathway. Western‐blot analysis of tumor tissues showed that the expression levels of AIF, HtrA2, Smac/Diablo, and Cyto‐C in the Ad‐Vp3 + BAPTA‐AM and Ad‐Vp3 groups were significantly higher than the BAPTA‐AM, Ad‐Mock, and PBS groups (*p* < 0.05). However, the expression levels of Smac/Diablo and Cyto‐C in the Ad‐Vp3 + BAPTA‐AM group were significantly lower than those in the Ad‐Vp3 group (Figure 6A) (*p* < 0.01). The trends of variation for the expression levels of AIF, HtrA2, Smac/Diablo, and Cyto‐C according to the immunohistochemical data (Figure 6B) were similar to that shown by western blotting. Collectively, these in vivo results showed that Apoptin caused significant changes in the expression of AIF, HtrA2, Smac/Diablo, and Cyto‐C and that BAPTA‐AM could reduce the expression of Smac/Diablo and Cyto‐C in Apoptin‐treated tumor tissues.

## 4 DISCUSSION

The endoplasmic reticulum is a highly dynamic organelle that plays a key role in homeostasis. The homeostatic balance created by the ER can, however, be disrupted by genetic and environmental damage, thus resulting in ER stress. In order to restore dynamic balance, the ER stress pathway is triggered and produces increased levels of several stress‐related proteins, including PERK,^22^, ^23^, ^24^ IRE1^25^, ^26^ and ATF‐6.^27^, ^28^, ^29^ Collectively, these proteins lighten the load on the ER by reducing misfolding and helping to avoid the accumulation of unfolded proteins. In this study, we found that the expression levels of proteins in the ER stress pathway, including PERK and ATF‐6, were significantly higher in HepG‐2 cells infected with Ad‐Vp3 than cells infected with Ad‐Mock when tested at 12 and 24 h post‐infection. These changes were caused by ER stress. However, the expression levels of these proteins in Ad‐Vp3‐infected cells were significantly lower than those in the Ad‐Mock‐infected cells, at 48 and 72 h post‐infection. These changes might be caused by ER stress injury. Electron microscopy also revealed that the ER in HepG‐2 cells had incurred significant structural damage when assessed at 48 h post‐infection. Collectively, these results indicated that the structure and function of the ER were both damaged by strong and persistent ER stress, thus resulting in the increased expression of ER‐related proteins; subsequently, these ER‐related proteins showed a significant decrease in expression. In conclusion, these data indicate that Apoptin can cause ER stress injury in HepG‐2 cells by inducing strong and persistent ER stress.

From one perspective, ER stress and injury can both cause an increase in intracellular Ca^2+^ levels, thus resulting in a Ca^2+^ imbalance.^30^, ^31^, ^32^ The levels of Ca^2+^ in Ad‐Vp3‐infected cells were significantly higher than those in Ad‐Mock‐infected cells and non‐infected HepG‐2 cells at 24, 36, and 48 h post‐infection. This was caused by strong and lasting ER stress, followed by ER stress injury. However, Ca^2+^ levels in the Ad‐Vp3‐infected cells were significantly lower than that in the control groups at 72 h post‐infection. This was because the Ad‐Vp3‐infected cells underwent a significant extent of apoptosis. In the present study, Apoptin triggered the ER stress pathway in the ER, thus increasing the expression levels of PERK, IRE1, and ATF‐6. Consequently, Apoptin can induce a strong and lasting ER stress and Ca^2+^ imbalance.

An imbalance in the intracellular concentration of Ca^2+^ can affect the mitochondrial membrane potential^33^ and subsequently induce the release of pro‐apoptotic proteins from the mitochondrial membrane mesenchyme.^34^ Several pro‐apoptotic proteins can be released from the mitochondria, including AIF, HtrA2, Endo G, ARTS, Smac/Diablo, and Cyto‐C. AIF is a flavoprotein and represents the main protein responsible for Caspase‐independent apoptosis. HtrA2 and Caspase are known to competitively bind Inhibitors of Apoptosis Proteins (IAP).^35^, ^36^ The interaction between these pro‐apoptotic proteins and IAP can relieve the inhibition of caspase by IAP, thus allowing caspase to become activated and then induce apoptosis. Endo G is known to migrate to the nucleus and trigger cell apoptosis by causing DNA fragmentation.^37^, ^38^, ^39^ ARTs are known to mediate various pro‐apoptotic stimuli to induce apoptosis. The function of Smac/Diablo is the same as HtrA2.^36^, ^40^ In vitro, we found that the mitochondrial membrane potential of Ad‐Vp3‐infected cells was significantly lower than that in Ad‐Mock‐infected cells and non‐infected HepG‐2 cells at 24 post‐infection. Furthermore, compared with all other time points, the mitochondrial membrane potential at 36 h post‐infection, was also significantly reduced in the cells infected with Ad‐Vp3. We speculate that this result might be caused by an abnormal change in intracellular Ca^2+^ level. To analyze whether Ca^2+^ imbalance affects the abnormal changes of mitochondrial membrane potential mediated by Apoptin in HepG‐2 cells, Ad‐Vp3 infected cells were further treated with BAPTA‐AM. These results showed that the imbalance of Ca^2+^ induced by Apoptin could reduce the mitochondrial membrane potential and induce apoptosis. In vivo, the regulation of Ca^2+^ imbalance reduced the expression levels of Smac/Diablo and Cyto‐C in the Ad‐Vp3+ BAPTA‐AM group. This result showed that Apoptin might increase the expression levels of Smac/Diablo and Cyto‐C to inhibit the proliferation of HepG‐2 cells in tumor‐bearing nude mice by inducing Ca^2+^ imbalance.

In this study, we found that Apoptin induced strong and lasting ER stress and thus caused ER stress injury. These events subsequently led to an imbalance of intracellular Ca^2+^, a reduction in mitochondrial membrane potential, a significant extent of damage in the mitochondrial structure, and an increase in the expression levels of Smac/Diablo, and Cyto‐C, by triggering the mitochondrial apoptotic pathway in HepG‐2 cells. However, it is not clear how Apoptin induces ER stress. We speculate that Apoptin‐induced ER stress may be related to Apoptin‐induced G2/M phase arrest.^41^, ^42^ G2 phase involves the synthesis of many RNAs and proteins. Therefore, G2/M phase arrest may cause an accumulation of unfolded or misfolded proteins, thus triggering the unfolded protein response (UPR), leading to ER stress. Previous studies have not reported the specific relationship between G2/M phase arrest and UPR/ER stress. It is clearly evident that we now need to be able to elucidate the specific relationships between these three factors. By investigating the relationships between G2/M phase arrest, UPR, and ER stress, we may be able to enhance our understanding of the role of Apoptin‐induced ER stress in Apoptin‐induced apoptosis. Therefore, in the future, we will use miRNA interference to further investigate the relationship between cell cycle G2/M phase block and unfolded protein response by detecting cell cycle‐related proteins (securin, cyclin B, CHK1, and p‐CHK1) and folded protein reaction‐related proteins (IRE1/XBP‐1s, ATF6, and PERK/eIF2α).

## AUTHOR CONTRIBUTIONS

**Xiaoyang Yu:** Data curation (equal); formal analysis (equal); investigation (equal); methodology (equal); writing – original draft (equal). **Tongxing Wang:** Data curation (equal); formal analysis (equal); investigation (equal); methodology (equal); writing – original draft (equal). **Yue Li:** Data curation (equal); formal analysis (equal); investigation (equal); methodology (equal); writing – original draft (equal). **YIquan Li:** Investigation (equal). **Bing Bai:** Investigation (equal). **Jinbo Fang:** Investigation (equal). **Jicheng Han:** Investigation (equal). **Shanzhi Li:** Investigation (equal). **Zhiru Xiu:** Investigation (equal). **Zirui Liu:** Investigation (equal). **Xia Yang:** Investigation (equal). **Yaru Li:** Investigation (equal). **Guangze Zhu:** Investigation (equal). **Ningyi Jin:** Investigation (equal). **Chao Shang:** Writing – review and editing (equal). **Xiao Li:** Writing – review and editing (equal). **Yilong Zhu:** Data curation (equal); formal analysis (equal); investigation (equal); methodology (equal); writing – original draft (equal).

## FUNDING INFORMATION

This study was sponsored by the Changchun Science and Technology Development Plan Project (No.Reference: 21QC01), the Science and Technology Research Planning Project of Jilin Provincial Department of Education (No.Reference: JJKH20220887KJ), the National Natural Science Foundation of China (No.Reference: 82151221), the Jilin Traditional Chinese Medicine Science and Technology Project in 2022 (No.Reference: 2022133), the Jilin Science and Technology Development Plan Project (No.Reference: 20220508075RC) and the Young Scientist Program Training Program of Changchun University of Chinese Medicine (No.Reference: QNKXJ2‐2021ZR11).

## CONFLICT OF INTEREST

None of the authors have any conflicts of interest to declare.

## ETHICS APPROVAL AND CONSENT TO PARTICIPATE

The animal experiment protocols were approved by the Institutional Animal Care and Use Committee of the Changchun University of Chinese Medicine (Approval No. 2021078).

## INFORMED CONSENT

Informed consent was obtained from all participants included in the study.

## RESEARCH INVOLVING HUMAN PARTICIPANTS AND/OR ANIMALS

The animal experimental protocols were approved by the IACUC of the Changchun Veterinary Research Institute, Chinese Academy of Agricultural Sciences (Changchun, China) (10ZDGG007). The manuscript does not feature human clinical studies or patient data.

## Data Availability

Data sharing is not applicable to this article as no new data were created or analyzed in this study.
